# WWOX modulates the ATR-mediated DNA damage checkpoint response

**DOI:** 10.18632/oncotarget.6571

**Published:** 2015-12-12

**Authors:** Mohammad Abu-Odeh, Nyla A. Hereema, Rami I. Aqeilan

**Affiliations:** ^1^ Lautenberg Center for Immunology and Cancer Research, IMRIC, Hebrew University-Hadassah Medical School, Jerusalem 91120, Israel; ^2^ Department of Pathology, Immunology and Medical Genetics, Wexner Medical Center, Ohio State University, Columbus, OH 43210, USA; ^3^ Department of Molecular Virology, Immunology and Medical Genetics, Wexner Medical Center, Ohio State University, Columbus, OH 43210, USA

**Keywords:** WWOX, genomic instability, ITCH, ATR, common fragile sites

## Abstract

For many decades genomic instability is considered one of the hallmarks of cancer. Role of the tumor suppressor WWOX (WW domain-containing oxidoreductase) in DNA damage response upon double strand breaks has been recently revealed. Here we demonstrate unforeseen functions for WWOX upon DNA single strand breaks (SSBs) checkpoint activation. We found that WWOX levels are induced following SSBs and accumulate in the nucleus. WWOX deficiency is associated with reduced activation of ataxia telangiectasia and Rad3-related protein (ATR) checkpoint proteins and increased chromosomal breaks. At the molecular level, we show that upon SSBs WWOX is modified at lysine 274 by ubiquitination mediated by the ubiquitin E3 ligase ITCH and interacts with ataxia telangiectasia-mutated (ATM). Interestingly, ATM inhibition was associated with reduced activation of ATR checkpoint proteins suggesting that WWOX manipulation of ATR checkpoint proteins is ATM-dependent. Taken together, the present findings indicate that WWOX plays a key role in ATR checkpoint activation, while its absence might facilitate genomic instability.

## INTRODUCTION

Genomic instability, one of the cancer hallmarks, plays critical roles both in tumor initiation and progression. It is characterized by accumulation of chromosomal changes ranging from mutations within the DNA sequence to structural abnormalities [1]. In eukaryotic cells the most common causes of genomic instability are failure of DNA replication and DNA-damage response (DDR), which are increased by external genotoxic agents and/or cellular pathologies [2]. The cells can use a repertoire of repair mechanisms during all stages of the cell cycle to preserve the genome from the mutagenic action of genotoxic agents and to guarantee faithful chromosome duplication and transmission to the daughter cells [3]. The DDR is considered as one of the first lines of defense to safeguard against genomic instability. Following DNA damage the cells activate highly conserved kinase-based signaling network of tightly regulated events, including sensing of DNA damage, accumulation of DNA repair factors at the site of damage, and finally physical repair of the lesion [4]. Upon a severe damage the cells are facing the fate-decision: to undergo apoptosis or senescence or to live with mutated genome [5].

DNA single- and double-strand breaks (SSBs and DSBs, respectively) activate common and distinct checkpoints during the cell cycle. DSBs are cytotoxic and must be repaired in order to complete the cell-cycle [6]. They can be generated by exogenous agents such as ionizing radiation (IR) or by endogenously generated reactive oxygen species (ROS). Following IR, ATM (ataxia telangiectasia mutated) is activated to mediate repair through phosphorylation of several targets, like H2AX, KAP1, and CHK2 [6]. Consequently, DNA is repaired or if the damage is too severe, cells apoptose.

Another common form of DNA damage is SSBs. Although SSBs have milder effect than DSBs, they are toxic to the cell as they can block DNA replication and transcription. Accordingly, defects in SSB repair are associated with several hereditary neurodegenerative diseases [7]. Exogenously, SSB could be induced by (i) Ultraviolet radiation (UVR) which may cause cyclobutane-pyrimidine dimers (CPDs) as well as DNA strand breaks [8], (ii) the anti-tumor drug Hydroxyurea (HU), a potent inhibitor of ribonucleotide reductase that halts DNA replication through its effects on cellular deoxynucleotide pools [9], and (iii) Aphidicolin (APH), a specific and mild inhibitor of DNA polymerase, also known to induce chromosomal aberrations, specifically at common fragile sites (CFSs) [10, 11]. These treatments, among many others, lead to stalling of the replication fork which result in SSBs that can also, if not repaired, develop into DSBs. Upon DNA SSBs, the Serine/threonine-protein kinase Ataxia Telangiectasia and Rad3-related protein (ATR) senses the damage and activates its downstream target checkpoint kinase 1 (CHK1), leading to cell-cycle arrest in order to repair the damaged DNA [12]. It is well accepted that ATM is activated upon DSBs, but evidence suggests that ATM phosphorylation upon UVR is ATR-dependent [13, 14]. Moreover, in some circumstances there is interplay between ATM and ATR functions in order to maintain the integrity of the whole genome and in particular CFSs [15].

Recently we showed that the WW domain-containing oxidoreductase (WWOX) is involved in DSB repair [16]. WWOX physically interacts and supports efficient activation of ATM whereas WWOX deficiency results in reduced activation of ATM, inefficient phosphorylation of its substrates, and impaired DNA repair [16]. The *WWOX* gene, located at chromosome region 16q23.3-q24.1, spans the chromosomal CFS FRA16D. This gene encodes a 46kDa protein that contains two N-terminal WW domains, of which WW1 domain mediates the interaction with WWOX partners [17] and a central short-chain dehydrogenase/reductase domain that has been proposed to function in steroidogenesis [18, 19].

CFSs are chromosome structures that are particularly prone to breakage under conditions of replication stress [20]. Recently, CFSs have become of increasing interest in cancer research, as they not only appear to be frequent targets of genomic alterations in cancer progression, but also already in precancerous lesions [21, 22]. Despite growing evidence of their importance in disease development, most CFSs have not been investigated at the molecular level, and the consequences of fragile genes (non-coding or coding) is not well understood [23]. The facts that WWOX is induced and functionally associates with ATM upon DSBs argue against its passive role in tumorigenesis. To further learn about WWOX function upon DNA damage, we studied its response upon SSBs.

Early evidence suggested that WWOX transcript is downregulated following UVR however its protein levels stayed stable and only decreased after repeated exposures [24]. By contrast, murine WOX1 levels were shown to be induced early following UV light treatment both *in vitro* [25] and *in vivo* [26]. More recently, it has been shown that UV radiation rapidly induced WWOX accumulation in the nucleus within 10-30 min [27]. WWOX levels dropped back to normal after 24hr suggesting a role of WWOX in DDR upon SSBs induction [28]. Nevertheless, the molecular and cellular role of WWOX upon SSB is poorly understood.

Here we show a novel role for WWOX in activation of DNA-damage checkpoint following DNA SSBs induced by UVC, HU, and APH. We found that ATR-checkpoint activation by WWOX is ATM-dependent. Upon DNA SSBs, WWOX expression is induced, predominantly at the protein level. We also found that the ubiquitin-E3 ligase ITCH, which we recently demonstrated its physical interaction with WWOX [17], enhances WWOX ubiquitination, at lysine (K) 274, and stabilizes its protein following SSBs where it activates ATM and ATR. Importantly, targeted loss of WWOX enhances chromosomal breaks upon APH treatment. Our findings identify an important role for the tumor suppressor WWOX upon SSBs and suggest that its loss may drive genomic instability and provide an advantage for clonal expansion of neoplastic cells.

## RESULTS

### Effect of DNA single strand breaks on WWOX levels

**Figure 1:**
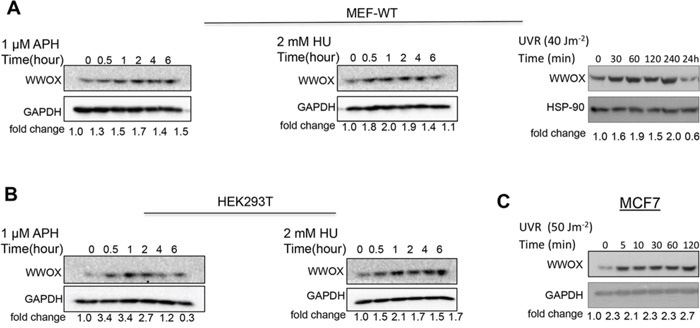
Induction of WWOX expression early after DNA-damage stimuli **A**. Immunoblot analysis of WWOX levels in early passage MEFs following treatment with 1 μM APH (left panel) or 2mM HU (medal panel) or 40 Jm^−2^ UV (right panel) for indicated time points. **B**. Immunoblot analysis of WWOX levels in HEK293T treated with 1 μM APH (left panel) or 2mM HU (right panel) for indicated time points. **C**. Immunoblot analysis of WWOX levels in MCF7 cells following treatment with UVC for indicated time points. Aphidicolin (APH), Hydroxyurea (HU), UV radiation (UVR). Equal loading was confirmed by probing with anti-GAPDH or HSP-90 specific antibody.

Very recently it has been reported that following DSBs WWOX levels are induced [16]. These results prompted us to determine whether induction of DNA SSBs has any effect on WWOX levels. To this end, SSBs were induced in primary non-tumorigenic MEFs using APH, HU and, and UVC and WWOX levels were assessed. Immunoblot analysis revealed that WWOX protein levels in early passage MEFs are induced following 30 min treatment with APH or HU or UVC (Figure 1A). A comparable induction was also seen in HEK293T cells (Figure 1B and Figure S1A). WWOX protein levels were also induced upon UVC treatment in MCF7 cells (Figure 1C and Figure S1B). We then examined if WWOX mRNA levels are also induced following DNA SSBs. We found that WWOX expression, as assessed by real-time PCR, was upregulated 2 hours after UVC exposure but did not change after HU or APH treatment (Figure S2) suggesting that induction of WWOX at early time points is postranslationally regulated. These results suggest that WWOX plays an important role upon SSBs in non-tumorigenic and tumorigenic cells.

### WWOX regulate DNA damage response (DDR) checkpoint proteins following SSBs

**Figure 2:**
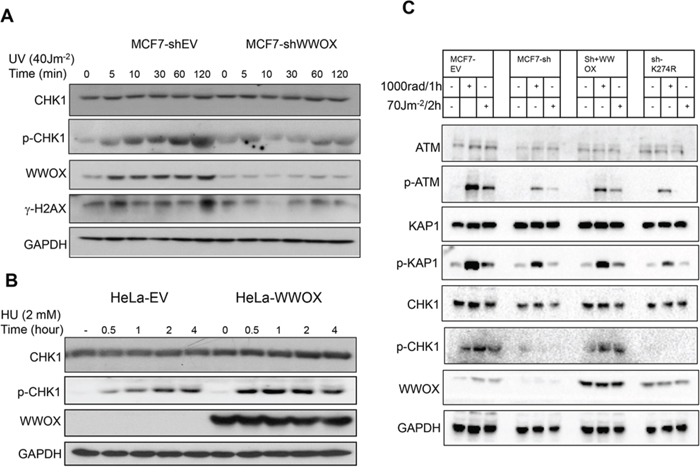
WWOX regulates DNA-damage response following SSBs **A.** MCF7-sh EV and MCF7-shWWOX cells were untreated or treated with UVC for the indicated time points. Whole cell lysate were analysed by immunoblot using specific antibody against CHK1, p-CHK1 (Ser296), WWOX, γ-H2AX (pSer139), and GAPDH. **B**. Immunoblot analysis of phospho-CHK1 activation in WWOX-overexpressing HeLa cells after HU treatment. HeLa-EV or HeLa-WWOX cells were treated with HU for the indicated times. Cell lysates were then probed with antibodies against CHK1, p-CHK1, WWOX and GAPDH. **C**. MCF7 depleted WWOX (MCF7-sh) cells were infected with lentiviral vector expressing EV, or WWOX or mutant WWOXK274R. Whole cell lysates were collected 1h following IR or 2h following UVC treatment then analyzed by immunoblotting using specific antibody against ATM, p-ATM (Ser1981), KAP1, p-KAP1 (pThr824), CHK1, p-CHK1 (Ser296), WWOX, and GAPDH.

Since WWOX is induced upon SSBs, we set out to determine whether its loss modulates DDR checkpoint proteins. Impaired DDR is one of the main causes of cancer development [3]. The main regulator of SSBs repair is ATR, which following DNA damage activates its downstream substrate, CHK1 [29]. Therefore, we set to examine activation of the ATR signalling pathway in MCF7 cells following depletion of WWOX and SSBs induction. MCF7 control cells (MCF7-shEV) showed an efficient accumulation of activated ATR target CHK1 (p-CHK1S296) and phosphorylated H2AX (γ-H2AX) following UVC treatment (Figure 2A). In contrast, MCF7-WWOX depleted cells (MCF7-shWWOX) show reduced activation of both proteins (p-CHK1 and γ-H2AX), suggesting a defect in the signalling pathway of ATR. On the other hand overexpression of WWOX in HeLa and KHOS cells, two WWOX-negative cells, led to improve CHK1 activation upon HU or UVC treatment (Figure 2B, Figure S3).

We then wondered whether restoration of WWOX in WWOX-depleted MCF7 cells could rescue the defect in ATR checkpoint proteins. By using lenti-viral vector we restored wild type WWOX or WWOXK274R, a mutated form of WWOX in which Lysine (K) 274 is ubiquitinated by the ubiquitin E3 ligase ITCH [17] after DNA DSBs [16], and stable clones were generated. Consistent with previous results and as expected, knockdown of WWOX in MCF7 cells attenuated checkpoint activation following DSBs (IR) and SSBs (UVC) (Figure 2C). Restoration of WT form of WWOX, but not WWOXK274R, into MCF7-shWWOX could rescue the defect in ATR checkpoint activation upon DNA damage (Figure 2C). To rule out that the relative reduced expression levels of WWOXK274R is responsible for the impaired checkpoint activation observed in Figure 2C, we generated new MCF7 clones expressing higher levels of WWOXK274R and examined their response. We found that even when WWOXK274R expression levels are higher, it could not restore proper protein checkpoint activation (Figure S4) suggesting that lysine 274 is not only important for WWOX stability but also necessary for WWOX signalling upon DNA damage. All together these findings indicate that WWOX regulates ATR checkpoint activation and that K274 plays a critical role in this function.

### WWOX modulates the G2/M checkpoints following DNA damage

DNA checkpoint activation is generally accepted as one of the critical components of cell survival after exposure to DNA damage. We hence investigated whether WWOX is involved in the regulation of the G2/M checkpoint after DNA damage. To this end, control and WWOX-depleted MCF7 cells were exposed to UVC and then labelled with anti-phospho-histone 3 (pH3), a marker for cells in the M phase. In contrast to control cells, which were readily arrested in G2, a significantly higher population of WWOX-depleted cells entered mitosis (Figure S5). These results suggest that WWOX loss is associated with defective G2/M cell-cycle checkpoint that could lead to genomic instability.

### WWOX deficiency enhances chromosomal breaks upon DNA damage

**Figure 3:**
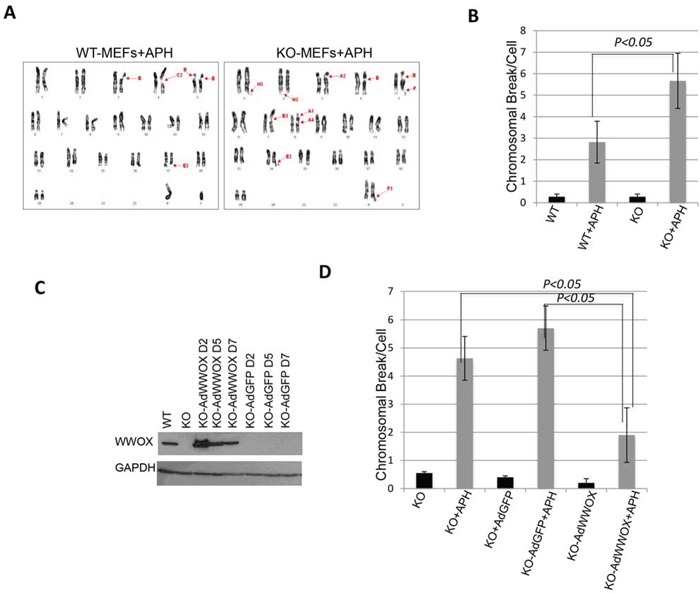
WWOX deficiency sensitizes cells to chromosomal breaks upon DNA damage **A.** Representative picture of karyotype analysis of wild type and *Wwox*-deficient MEFs following treatment with APH (0.2 μM) for 16 hours. Arrows indicated breaks or gaps. **B.** Average total chromosome gaps and breaks per cell (n=23 cell metaphases) in *Wwox*-deficient (KO, n=3) or wild type (WT, n=3) MEFs in the absence (black bars) or presence of 0.2 μM APH (grey bars). **C.** Immunoblot showing ectopic WWOX expression in KO MEFs whole cell lysates two days (D2), D5 and D7 following transduction with Ad-WWOX or Ad-GFP. Equal loading was confirmed by probing with an a-GAPDH-specific antibody. **D.** Average total chromosomal breaks in KO, KO-AdGFP, or KO-AdWWOX MEFs in the absence (black bars) or presence of 0.2 μM APH (grey bars). Error bars indicate SEM.

Since WWOX expression is induced early upon DNA damage while its loss is associated with impaired checkpoint activation, we set to examine whether its loss affects genome stability. To do so, we examined whether *Wwox*-deficient (KO) MEFs display increased chromosomal breaks following a mild DNA replication stress using APH. To this end, wild-type (WT) and KO-MEFs were treated with low doses of APH and the number of chromosomal breaks was quantified using metaphase spreads. In the absence of APH, we observed some KO cells with spontaneous chromosomal breaks although not statistically significant relative to WT cells (Figure S6A). Treatment of WT MEFs with 0.2 μM APH induced an average of ∼2.8 ± 1 chromosomal breaks whereas 5.7 ± 1.7 breaks were detectable in KO-MEFs (*P<0.05*) (Figure 3A, 3B, Figure S6A). Since low doses of APH are known to induce CFS instability, we determined whether WWOX deficiency leads to increased chromosomal breaks at CFSs. Chromosomal banding showed that most chromosomal breaks observed were indeed in CFSs (Table S1). To further validate WWOX importance for chromosomal instability, we depleted WWOX expression in WT MEFs and examined their sensitivity to APH treatment. We found that WWOX knockdown (KD) is indeed associated with increased chromosomal breaks (Figure S6B). Next, we determined whether re-expression of WWOX in KO-MEFs would rescue this phenotype. KO-MEFs were infected with low titter of Adenoviral vector (Ad)-WWOX or Ad-GFP (Figure 3C) and 7-days later cells were treated with APH. Immunoblot analysis revealed comparable levels of WWOX expression in WT MEFs and KO-Ad-WWOX-infected MEFs (Figure 3C). Examination of metaphase spreads demonstrated that reconstitution of WWOX expression rescued the number of chromosomal breaks when compared to KO-Ad-GFP-infected and parental KO-MEFs (Figure 3D and Figure S6C). Notably, the number of breaks per cell in APH-treated KO-Ad-WWOX cells was comparable to that of WT cells (Figure 3D and Figure S6C). Overexpression of WWOX mutants, WWOX-WFPA (impaired in its interacting ability [16]) and WWOX-K274R did not reduce chromosomal breaks as intact WWOX did (Figure S6D); in fact, WWOXK274R expression exhibited a trend of increased chromosomal breaks (Figure S6D). These results further suggest that WWOX maintains chromosomal stability and that its deficiency renders the chromosomes less stable.

### WWOX accumulation and ubiquitination following DNA damage

**Figure 4:**
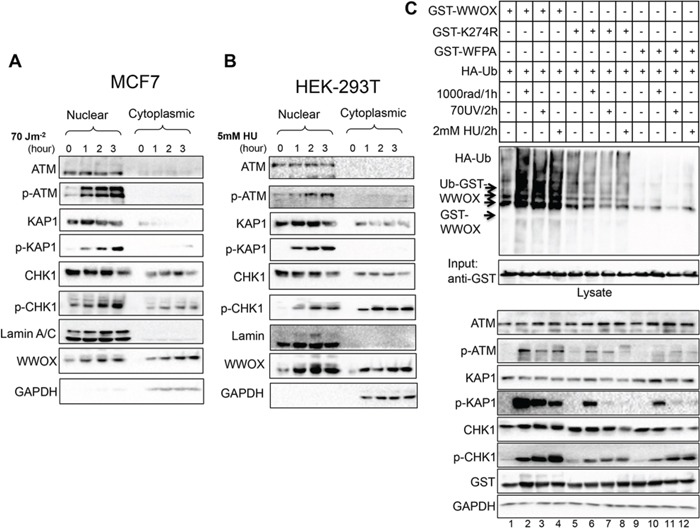
WWOX accumulation and ubiquitination in the nucleus following DNA SSBs **A.** MCF7 cells were untreated or treated with UVC as indicated in the figure for specific time points. Cells were then sub-fractioned into nuclear and cytoplasmic fractions, then analyzed by immunoblotting using ATM, p-ATM (Ser1981), KAP1, p-KAP1 (pThr824), CHK1, p-CHK1 (Ser296), lamin, and GAPDH. **B.** HEK293T cells were untreated or treated with HU as indicated in the figure for specific time points. Cells were then sub-fractioned in to nuclear and cytoplasmic parts, then analyzed by immunoblot using the indicated antibodies. **C.** DNA damage enhances WWOX ubiquitination. HEK293T cells were transfected with HA-UB, GST-WWOX, GST-K274R, and GST-WFPA plasmids, 24h later the cells were treated with IR or UVC or HU as indicated then analyzed by immunoblotting using antibodies against p-ATM (Ser1981), KAP1, p-KAP1 (pThr824), CHK1, p-CHK1 (Ser296), GST (WWOX) and GAPDH. GST pull-down was performed and the pull down complex was blotted using anti-HA, and anti GST.

So far our data indicate that WWOX is important for proper DNA damage checkpoint activation and chromosomal stability. We next set to address if induction of DNA SSBs is associated with nuclear accumulation of WWOX as WWOX is commonly localizes in the cytoplasm. Nuclear and cytoplasmic fractions were purified from damaged and undamaged MCF7 and HEK293T cells and analyzed for WWOX expression and checkpoint activation. GAPDH and Lamin A/C expressions in the cytoplasmic and nuclear fractions, respectively, were used to confirm successful subcellular fractionation. Following DNA damage, we observed both nuclear and cytoplasmic accumulation of WWOX (Figure 4A, 4B).

Several lines of evidence suggest that following DNA damage WWOX undergoes posttranslational modification, mainly ubiquitination [16]. In fact, we recently demonstrated that WWOX is a target of K63-linked ubiquitination resulting in its stabilization and nuclear accumulation [17]. Since WWOX levels are increased upon SSBs, we set to examine whether UVC or HU treatment enhances its ubiquitination. To that end, HEK293T cells were transfected with HA-Ubiquitin and GST-WWOX or GST-WWOXK274R or GST-WWOXWFPA. Twenty-four hours post transfection, cells were treated with UVC or HU. As positive control, we treated cells with IR and found that wild type WWOX ubiquitination is induced, as expected (Figure 4C upper panel line 1 vs. 2), but not the mutated form WWOXK274R (line 5 vs. 6) or WWOXWFPA (line 9 vs. 10). Interestingly we found that WWOX also underwent ubiquitination following both UVC and HU treatment relative to control untreated cells (Figure 4C, upper panel line 1 vs. 3 and 4) but not the mutant form of K274R (line 5 vs. 7 and 8) or WFPA (line 9 vs. 11 and 12). These findings demonstrate that WWOX is ubiquitinated and its levels are induced following induction of SSBs.

### WWOX associates with ATM following SSBs

**Figure 5:**
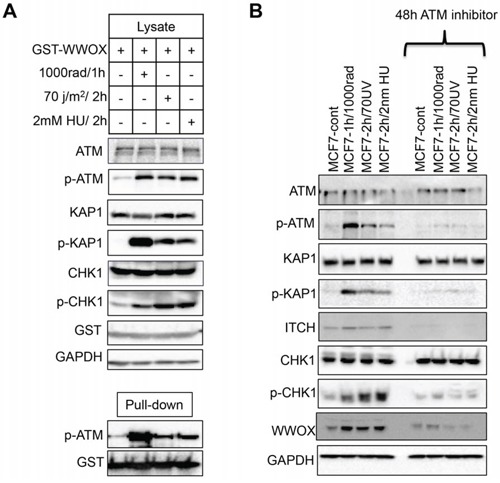
WWOX modulation of ATR checkpoint is ATM-dependent **A.** WWOX associates with p-ATM following induction of DNA SSBs. HEK293 cells were transfected GST-WWOX. At 24 hours, cells were treated with IR (10Gy for an additional hour) or UV ((70 J/m2) or HU (2mM) for an additional 2 hours). Cells were then subjected to GST-pulldown and Lysates were blotted against ATM, p-ATM (Ser1981), KAP1, p-KAP1 (pThr824), CHK1, p-CHK1 (Ser296), GST (WWOX) and GAPDH. Pulled-down complexes were blotted with anti p-ATM (Ser1981) and anti-GST (WWOX). **B.** MCF7 cells were cultured in duplicates, first duplicate were left untreated ore treated with IR or UVC or HU, the second duplicate were treated first with ATM inhibitor KU-55933 for 48h then were treated as first duplicate as indicated in the figure. Whole cell lysates were analysed by western blot using antibodies against ATM, p-ATM, KAP1, p-KAP1, ITCH, CHK1, p-CHK1, WWOX, and GAPDH as loading control.

Induction of DSBs leads to physical and functional interaction between WWOX and p-ATM contributing to efficient DDR [16]. We therefore set out to determine whether this interaction is also important for WWOX function upon SSBs. We first examined whether WWOX interacts with ATM upon SSBs. To this end, HEK293T cells were transfected with GST-WWOX and 24hs later the cells were treated with IR or UVC or HU and GST pull-downs were performed. As expected WWOX interacted with p-ATM following IR, but surprisingly, WWOX also associated with p-ATM following SSBs (Figure 5A). This result may suggest that the molecular mechanism by which WWOX manipulates ATR-checkpoint might be ATM-dependent.

### WWOX modulates ATR checkpoint is ATM-dependent

Our findings so far indicate that WWOX is induced following SSBs to accumulate in the nucleus and associate with p-ATM and that its deficiency impairs ATR-checkpoint activation. These observations prompted us to question how WWOX manipulates ATR checkpoint following DNA damage. Cumulative evidence indicates that ATM becomes phosphorylated upon UVR and this activation is ATR-dependent [13, 14]. To check whether this is indeed the circumstance in our case, MCF7 cells were untreated or treated with IR or UVC or HU, for the indicated time points in the presence or absence of ATM inhibitor (KU-55933). Whole cell lysates were prepared and analyzed by immunoblotting. As expected, we found that WWOX levels are induced following IR, UVC and UH (Figure 5B). Surprisingly, following ATM inhibition WWOX levels were significantly reduced. Moreover, ATR downstream target p-CHK1 levels as well as ATM and its targets KAP1 and ITCH, were also reduced following ATM inhibition (Figure 5B). This finding indicates that WWOX modulation of ATR checkpoint response might be ATM-dependent.

## DISCUSSION

Our study identified WWOX as an important player in DDR upon DNA SSBs. We showed that WWOX levels are induced upon HU, UVC and APH treatment, likely due to protein ubiquitination-mediated by ITCH. This induction is associated with increased nuclear accumulation where WWOX modulates ATR-checkpoint activation. Deficiency of WWOX enhances APH-mediated chromosomal breaks, a phenotype that could be rescued by ectopic expression of intact WWOX. Altogether, these observations indicate a direct function of WWOX in response to DNA SSBs.

The WWOX protein is a tumor suppressor that is lost or under-expressed in a wide variety of cancers, including breast, prostate, ovarian, and lung [17, 30–34]. Depletion of WWOX may occur through hemi- or homo-zygous deletions or by epigenetic alterations such as methylation of its promoter [35]. Emerging findings support the function of *WWOX* as a tumor suppressor: (i) overexpression of WWOX in WWOX-negative cancer cells reduces cell growth and suppresses tumor growth in immunodeficient mice [36–38]; (ii) *Wwox*-mutant mice showed higher incidence of spontaneous and chemically-induced tumors [39, 40]; (iii) WWOX molecularly regulates several cellular processes implicated in tumor initiation and/or progression [37, 41]. Our study further indicate that the molecular function of WWOX includes regulation of DDR checkpoint proteins that if impaired result in chromosomal instability. In particular, WWOX loss or depletion attenuates ATR-checkpoint activation and cell-cycle arrest. We show that under conditions of SSBs induction, WWOX physically interacts with p-ATM and mediates its activation. When ATM function is pharmacologically hampered, WWOX function is hindered and ATR signalling pathway is inhibited. This is likely mediated through reduced catalytic activity of ITCH which has been recently shown to be a substrate of ATM [42, 43] that targets by itself WWOX [44]. All these observations set WWOX as an important player in the DDR and provide evidence that its loss contribute to the tumorigenesis process. These data might also suggest that WWOX is important for other ATR functions including its role in mediating response to replication stress. Nevertheless, WWOX localization in a CFS, which is largely believed to be hot spot for stalling replication fork raises valid question about its contribution to this process.

Recent evidence has suggested that CFSs harbor functional units, genes and histone marks, which play active roles in carcinogenesis [45]. Our findings presented here and previous observations argue that WWOX, gene products of FRA16D, functions as a tumor suppressor. This does not seem to be limited to WWOX only as other gene products of CFSs have been linked with tumor suppressor functions. Work from the Huebner lab on the *FHIT* gene, spanning FRA3B, indeed supports a central role of the FHIT protein in genomic integrity and maintaining the thymidine triphosphate pool levels (reviewed in [46]). Products of FRA6E, FRA8I and FRA15A have been also associated with genome integrity and tumor suppression (reviewed in [47]). Altogether, these observations suggest an interesting emerging role of these loci in cancer and that CFSs have other roles beyond being cis elements that are sensitive for DNA damage.

It is possible that vulnerability of FRA16D to replication stress functions as a “cis” sensor to DNA damage. Whether this alteration is associated with impaired WWOX protein (trans) expression/function is unknown. Intriguingly, most of the breaks in *WWOX* that are documented in TCGA database are focally located in intron 8. How this affects WWOX mRNA splicing and protein expressions are largely unknown. Our results clearly demonstrate that WWOX protein levels are increased upon DNA damage to support efficient DDR ([16] and this work). It would be of great interest to dissect whether intron 8 of WWOX harbors functional elements, such as non-coding RNA or histone marks, which might be important for its function. It is possible that these loci functions as sensors for DNA damage but emerging data suggest that their associated genes and protein products could also be transducers and/or effectors in the DDR.

**Figure 6:**
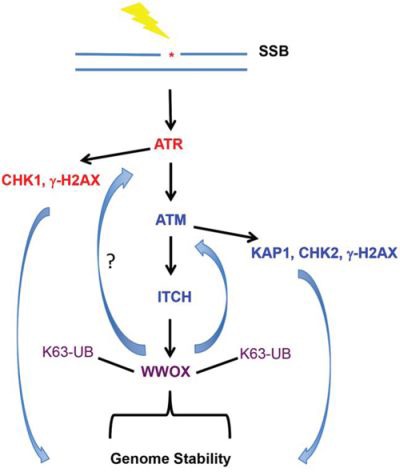
Summary illustration of WWOX action upon DNA SSB Induction of SSBs activates ATR-CHK1 pathway. ATM is activated to mediate ATM-CHK2 response. ATM could phosphorylate ITCH that mediates WWOX ubiquitination and stabilization which together with p-ATM maintains efficient DDR and guard stability of the genome. Inhibition of ATM renders WWOX less active (not shown). Activation of WWOX acts in a feed-forward loop to maintain ATM efficient DDR (16) and possibly ATR checkpoint activation.

Our results show that WWOX levels accumulate after DNA damage. In the nucleus, we found that even after SSBs, WWOX binds p-ATM and modulates the ATM checkpoint activation as well as the ATR signaling pathway. Since ATM inhibition was associated with reduced ATR activation, we believe that WWOX modulation of ATR function is ATM-dependent (Figure 6). Nevertheless, we cannot exclude that WWOX could regulate other players in the ATR signaling pathway. It is also known that ATM inhibition results in pan changes that could affect DDR signalling; i.e increased levels of tumor suppressor *ARF* [48].

WWOX levels are also increased in the cytoplasm, which suggests that WWOX might have important cytoplasmic functions upon DNA damage. In fact, it has been shown that WWOX binds a number of proteins in the cytoplasm and regulates their functions. One relevant example is p73 with which WWOX interacts in the cytoplasm and mediates its transactivation-independent apoptosis [49]. Murine WOX1 was also shown to be essential for UVB-induced apoptosis *in vivo*; UVB promotes WOX1-Tyr33 phosphorylation and accumulation to enhance normal keratinocyte differentiation and cell death [26]. The fact that WWOX is induced upon DSBs and SSBs and that its deficiency is associated with increased chromosomal/genomic instability indicates its critical role in the DDR. We propose that only when WWOX is lost, due to negative selection pressure, cells lose another level of its “brake” system contributing to tumor initiation and/or progression.

DNA breaks, both SSBs and DSBs, can be induced by a wide variety of agents including extrinsic UVR but also intrinsic ROS and oncogene activation. How the later affects WWOX expression is poorly understood and is currently being addressed. It will be interesting to use real-time imaging technology [50–52] to follow the DDR and repair in WWOX-deficient and sufficient cells. The single and double strand breaks are not only relevant for cancer as emerging evidence links DNA damage with neurological and metabolic disorders [19]. Recent observations have also associated WWOX with such modalities [53, 54]. Future studies in this direction should further deepen our understanding of WWOX functions in the DDR related to cancer and likely other pathologies.

## MATERIALS AND METHODS

### Cell culture and transfection

HEK293T, MCF7 and KHOS were cultured in RPMI, early passage mouse embryonic fibroblasts (MEF) and HeLa were cultured in DMEM supplemented with heat inactivated Fetal Bovine Serum (FBS) (10%) (Gibco). Transient transfections were performed using Mirus TransLTi (Mirus Bio LLC) according to the manufacturer.

### RNA extraction and mRNA level quantification

Total RNA, cDNA preparation and Real Time PCR were done as previously described [16].

### Karyotype analysis

MEF isolated from *Wwox*-deficient mice (MEF−/−) and wild type (MEF+/+) was treated with 0.2 μM aphidicolin for 16 hours. Colcemid (0.1 μg/ml) was added for 2 hours, a chromosome spread was performed using a hypotonic solution (0.075 M KCL) and then cells were fixed with a mixture of methanol and acetic acid (3:1). These cells were then spread on slides, air-dried, and stained with trypsin-Wright stain. Chromosomal breaks (of at least 20 metaphase spreads) were visualized and quantified using light microscope.

### G2/M checkpoint analysis

Cells were untreated or treated with UVC (50 jm^−2^). At 6, or 9h after treatment, cells were harvested and washed with PBS, and then fixed with 1% formaldehyde for 10 min at 37°C. Cells were chilled on ice for 1 min and then permeabilized with 90% methanol at −20°C overnight. The fixed cells were washed with PBS and blocked with incubation buffer (0.5% BSA in PBS) for 30 min. The cells were stained with anti-phospho histone H3 (S10) Alexa Fluor 647-conjugated antibody (Cell Signaling Technology) at 1:100 dilution in incubation buffer for 1 h in dark at room temperature; afterward cells were washed and resuspended in PBS containing 50 mg/mL PI. At least 10,000 cells were analyzed by FACScan.

### GST-pull down

GST-pull down was performed as previously described [16, 17]. In brief, HEK293T cells were transfected with the indicated plasmids and 24h post transfections cells were untreated or treated with DNA damaging agents. Total lysates were prepared and incubated with GST-beads at 4°C for 2 hours, then beads were washed and bound protein was eluted and analysed by immunoblot for the indicated proteins.

### Cellular fractionation

Nuclear and cytoplasmic fractionations were performed as previously described[16].

### List of antibody

Chk1 (A300-162A) and p-Chk1 (S296) (2349S), p-Histone H3 (S10) (D2C8)(Alexa®647), p-Histone H2A.X (S139) (20E3, 9718S), ATM (pS1981) (2152-1) (Cell Signaling. Danvers, MA), phospoho KAP-1 (S824) (A300-767A) and KAP1 (Cat # A300-274A), anti-ATM (A300-136A), (Bethyl, Montgomery, TX), Gout Polyclonal anti-WWOX antibody (a gift of Dr. Kay Huebner), anti-GAPDH mouse mAB (CB1001), anti-HSP90 rabbit (Cat # CA1016) (CALBIOCHEM, Billerica, MA), anti-Lamin A/C (N-18, Santa Cruz Biotechnology. Dallas, TX).

## SUPPLEMENTARY FIGURES AND TABLE




