# Supplementary material for: WWOX modulates the ATR-mediated DNA damage checkpoint response
Source: Oncotarget. 2015 Dec 12;7(4):4344–55. doi: 10.18632/oncotarget.6571 (PMC4826209; doi:10.18632/oncotarget.6571)
Supplement: Supplementary file 1 [file oncotarget-07-4344-s001.pdf]

SUPPLEMENTARY FIGURES AND TABLE

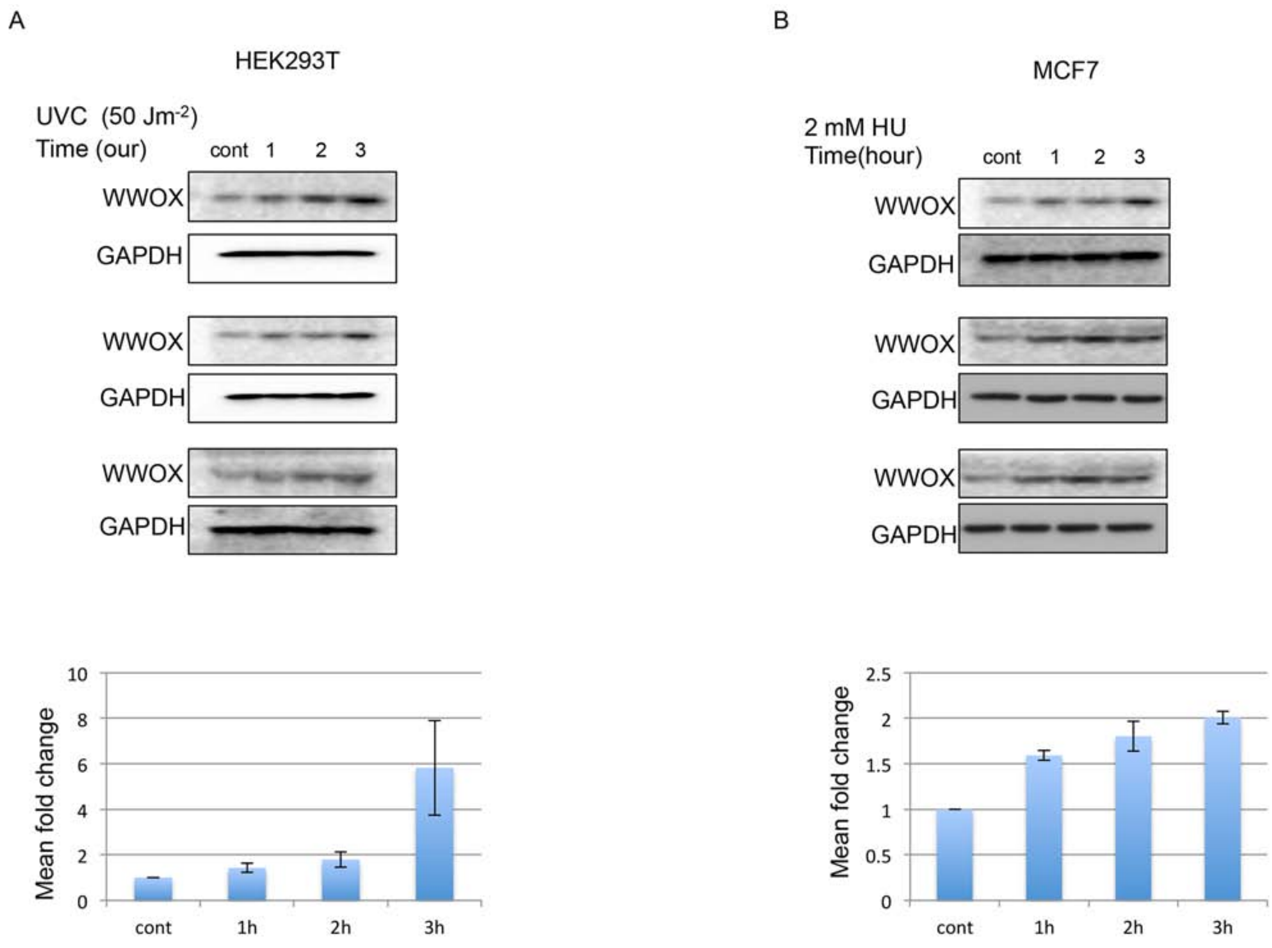

Supplementary Figure S1: Induction of WWOX expression early after DNA damage stimuli. A. Immunoblot analysis of WWOX levels in HEK293T treated with UVC for indicated time points. Three biological repeats are shown. **B.** Immunoblot analysis of WWOX levels in MCF7 cells following treatment with HU for indicated time points. Three biological repeats are shown. Below each panel (for A and B), mean fold change ± SEM of protein levels in each point from the three different experiments is shown.

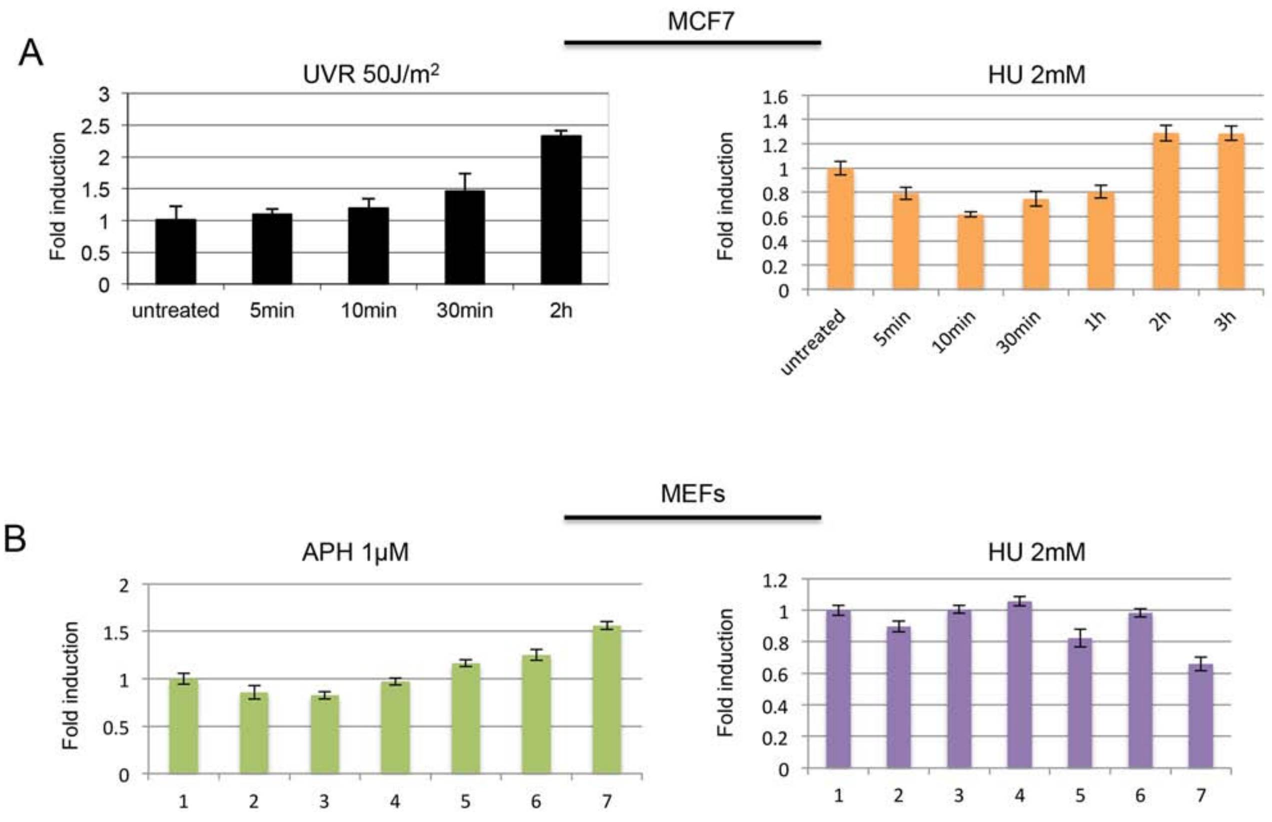

Supplementary Figure S2: Real-time analysis of *WWOX*. A. Real-time PCR analysis of *WWOX* in MCF7 following UVR (left panel) and HU (right panel) treatment for the indicated time points. **B.** Real-time PCR analysis of *WWOX* in the MEFs cells following APH (upper panel) and HU (lower panel) treatment as indicated in the figure. SEM is indicated.

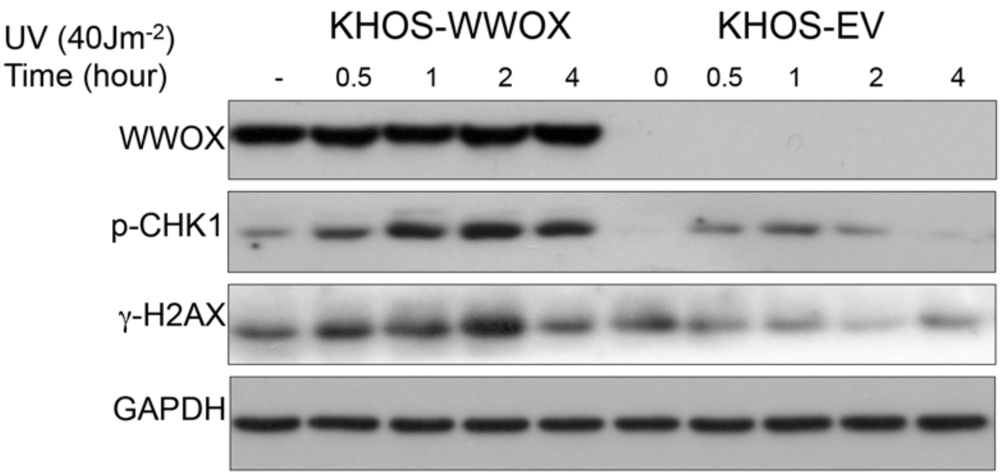

Supplementary Figure S3: Impaired checkpoint activation in *KHOS* cells. KHOS were infected with lentiviral victor expressing WWOX or scramble (EV), both cell types were untreated or treated with UVR (40Jm^{-2}) and whole cell lysate were collected at specific time point following UVR as indicated in the blot. Cell lysates were then analyzed by western blot using specific antibody against WWOX, p-CHK1 (Ser296) , γ-H2AX (pSer139) and GAPDH.

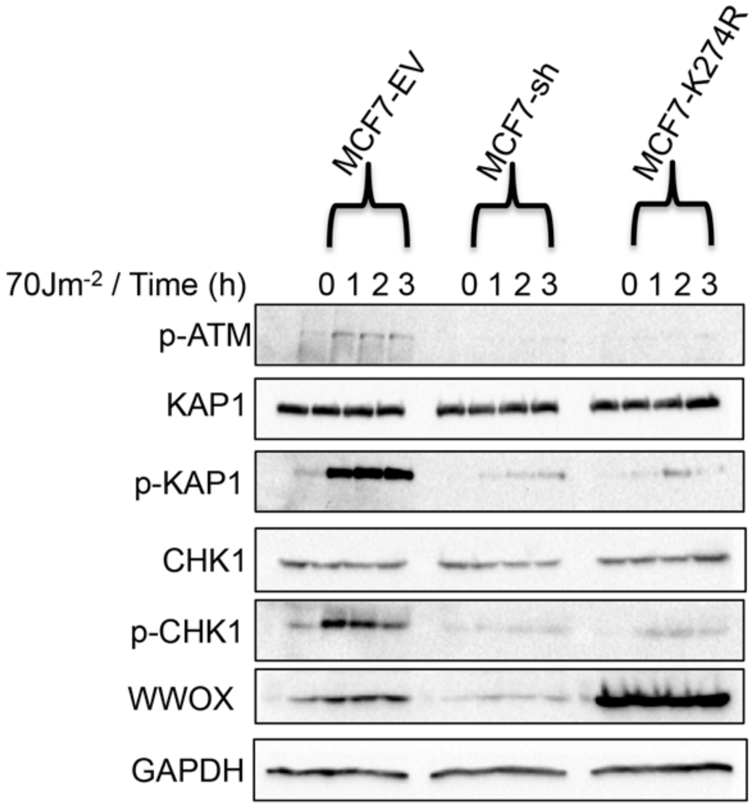

Supplementary Figure S4: Impaired checkpoint activation in MCF7 cells. MCF7-sh were infected with lentiviral vector expressing WWOX-K274R mutant or scramble (EV), both cell types were untreated or treated with UVC. Whole cell lysates were collected at specific time point following UVC as indicated. Cell lysates were then analyzed by western blot using specific antibody against WWOX, p- ATM, KAP1, p-KAP1, CHK1, p-CHK1 (Ser296) and GAPDH.

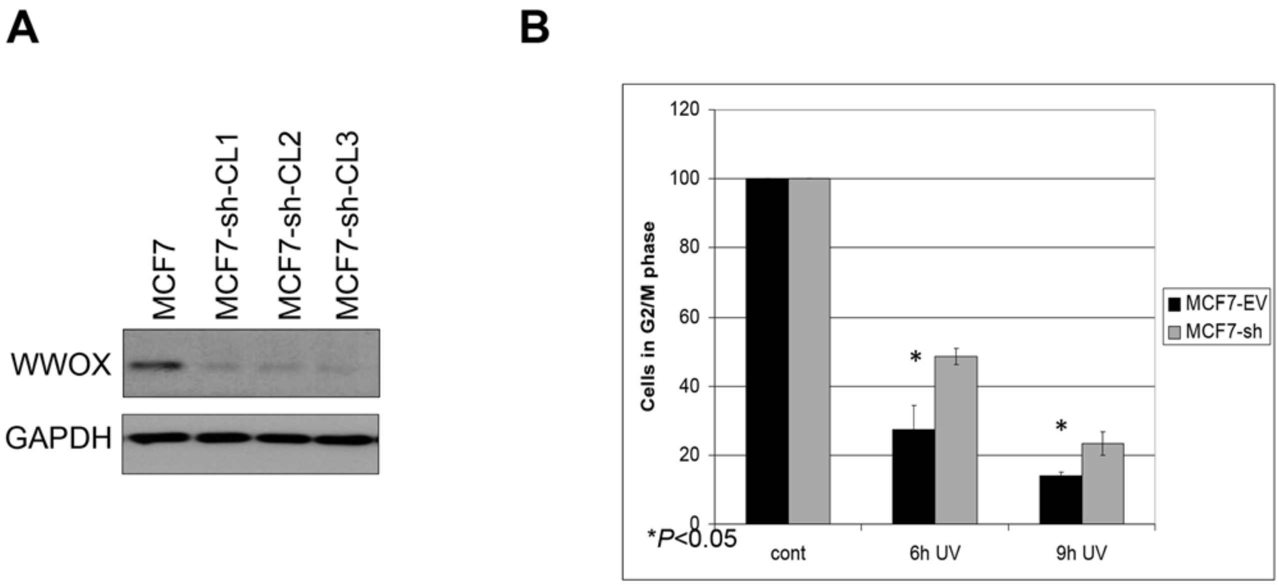

Supplementary Figure S5: WWOX modulates the G2/M checkpoints following DNA damage. A. MCF7 cells were infected with control or three different small hairpin RNA (shRNA) lentiviral expression vectors and selected with puromycine. Western blot of stable WWOXshRNA MCF7 expressing cells shows ~90% knockdown of WWOX. **B.** Control and WWOX knockdown MCF7 cells were untreated or treated with UVR (50jm^{-2}) before fixation. Cells in mitosis were determined by staining with phospho-histone H3 (pHH3) antibody followed by FITC-conjugated secondary antibody. The percentage of M-phase cells was determined by flow cytometry for pHH3. Values are expressed as relative pHH3 compared with untreated control cells. Data are presented as mean ± s.d.

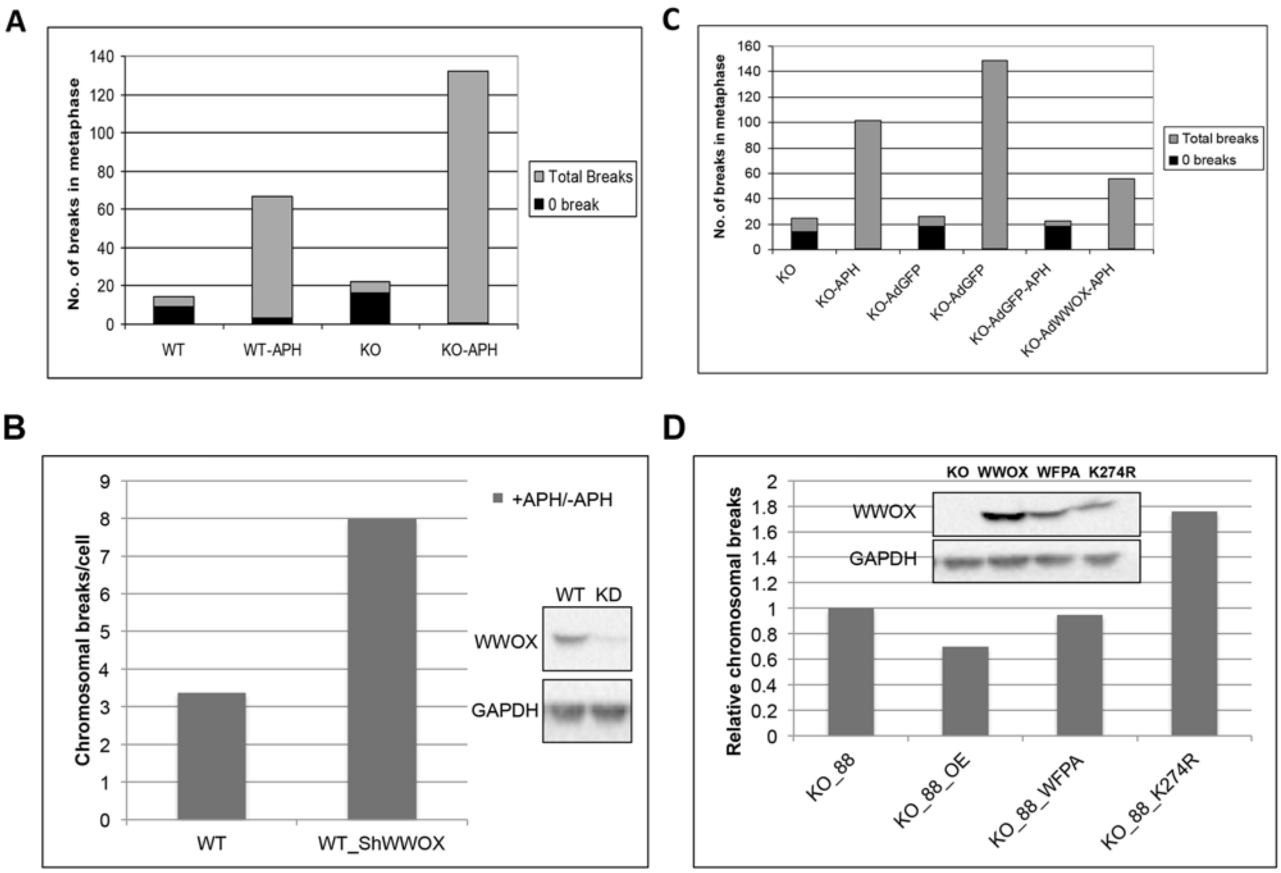

Supplementary Figure S6: A. Total chromosomal break analyses in wild type and *Wwox* deficient MEFs following treatment with aphidicolin (APH). Cell metaphases were prepared and number of breaks was quantified. Black bars represent cells containing no breaks (0 breaks) while gray bars represent total number of breaks in a give treatment. **B.** WWOX knockdown in *Wwox*- WT MEFs is associated with increased chromosomal breaks. Total number of chromosomal breaks, following treatment with 0.2 µM APH for 16 hours, in WT or WT-ShWWOX were determined as in A. Bars represent number of breaks in normal diploid cells (n=20) in presence of APH (+APH) relative to those in absence of APH (-APH). Immunoblot shows knockdown (KD) of WWOX in WT MEFs. **C.** WWOX overexpression in *Wwox*-deficient MEFs is associated with reduced number of chromosomal breaks. Total number of chromosomal breaks, following treatment with 0.2 µM APH for 16 hours, in KO, KO-AdGFP, or KO-AdWWOX MEFs were determined as in A. Black bars represent cells containing no breaks (0 breaks) while gray bars represent total number of breaks in a give treatment. **D.** Chromosomal break analysis in KO-MEFs overexpressing WWOX, WWOX-WFPA or WWOXK274R. Bars shows relative number of breaks (as in B) in the indicated cell metaphases (n=20) following treatment with 0.2 µM APH for 16 hour. Immunoblot shows expression of WWOX in the different clones.

Supplementary Table S1: Breakage sites in wild type and *Wwox*-deficient MEFs following treatment with Aphidicolin. Cells were treated as in Figure 3 and breaks and gaps location was determined.

See Supplementary File 1
