# Supplementary material for: WWOX modulates the ATR-mediated DNA damage checkpoint response
Source: Oncotarget. 2015 Dec 12;7(4):4344–55. doi: 10.18632/oncotarget.6571 (PMC4826209; doi:10.18632/oncotarget.6571)
Supplement: Supplementary file 2 [file oncotarget-07-4344-s002.pptx]

## Slide 1
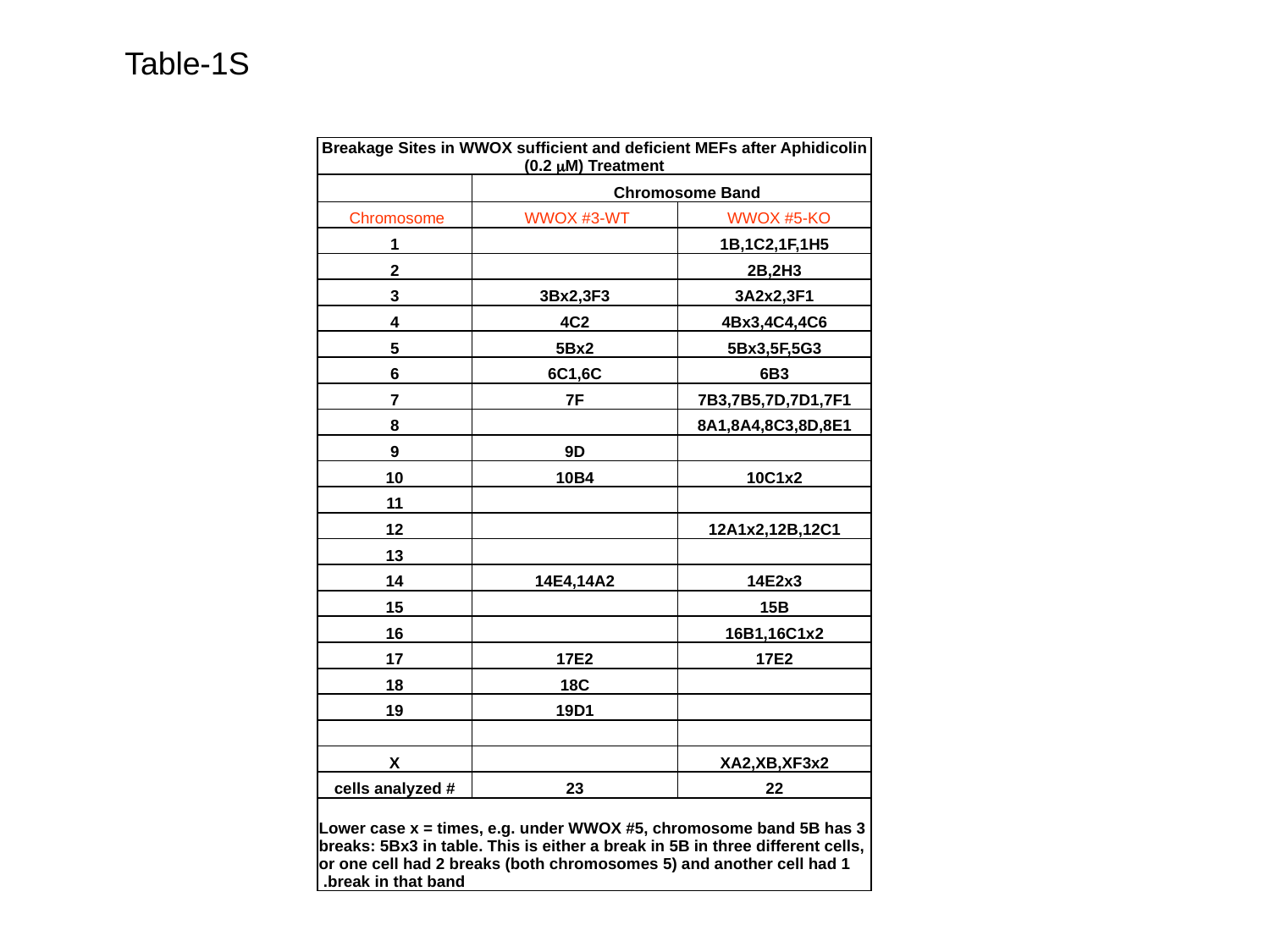

Table-1S
| Breakage Sites in WWOX sufficient and deficient MEFs after Aphidicolin (0.2 mM) Treatment | | |
| --- | --- | --- |
| | Chromosome Band | |
| Chromosome | WWOX #3-WT | WWOX #5-KO |
| 1 | | 1B,1C2,1F,1H5 |
| 2 | | 2B,2H3 |
| 3 | 3Bx2,3F3 | 3A2x2,3F1 |
| 4 | 4C2 | 4Bx3,4C4,4C6 |
| 5 | 5Bx2 | 5Bx3,5F,5G3 |
| 6 | 6C1,6C | 6B3 |
| 7 | 7F | 7B3,7B5,7D,7D1,7F1 |
| 8 | | 8A1,8A4,8C3,8D,8E1 |
| 9 | 9D | |
| 10 | 10B4 | 10C1x2 |
| 11 | | |
| 12 | | 12A1x2,12B,12C1 |
| 13 | | |
| 14 | 14E4,14A2 | 14E2x3 |
| 15 | | 15B |
| 16 | | 16B1,16C1x2 |
| 17 | 17E2 | 17E2 |
| 18 | 18C | |
| 19 | 19D1 | |
| | | |
| X | | XA2,XB,XF3x2 |
| # cells analyzed | 23 | 22 |
| Lower case x = times, e.g. under WWOX #5, chromosome band 5B has 3 breaks: 5Bx3 in table. This is either a break in 5B in three different cells, or one cell had 2 breaks (both chromosomes 5) and another cell had 1 break in that band. | | |
